# Supplementary material for: Modeling glioblastoma heterogeneity as a dynamic network of cell states
Source: Mol Syst Biol. 2021 Sep 16;17(9):e10105. doi: 10.15252/msb.202010105 (PMC8444284; doi:10.15252/msb.202010105)
Supplement: Supplementary file 6 — Source Data for Figure 5 [file MSB-17-e10105-s004.zip › Figure5A_sourcedata/GSEA_3017/nosigdb_stateB.GseaPreranked.1622795021725/RICHARDS_ZHONG_NATURE_2018_ASTROCYTES_UPREG.html]

Details for gene set RICHARDS\_ZHONG\_NATURE\_2018\_ASTROCYTES\_UPREG[GSEA]

|  || Dataset | state43017 |
| Phenotype | NoPhenotypeAvailable |
| Upregulated in class | na\_neg |
| GeneSet | RICHARDS\_ZHONG\_NATURE\_2018\_ASTROCYTES\_UPREG |
| Enrichment Score (ES) | -0.26290634 |
| Normalized Enrichment Score (NES) | -1.9663001 |
| Nominal p-value | 0.0 |
| FDR q-value | 0.013513701 |
| FWER p-Value | 0.049 |
Table: GSEA Results Summary

  

Fig 1: Enrichment plot: RICHARDS\_ZHONG\_NATURE\_2018\_ASTROCYTES\_UPREG      
 Profile of the Running ES Score & Positions of GeneSet Members on the Rank Ordered List

  

| PROBE | GENE SYMBOL | GENE\_TITLE | RANK IN GENE LIST | RANK METRIC SCORE | RUNNING ES | CORE ENRICHMENT || 1 | LRRN3 |  |  | 25 | 0.704 | 0.0151 | No |
| 2 | PMP22 |  |  | 70 | 0.576 | -0.0050 | No |
| 3 | GFAP |  |  | 132 | 0.487 | -0.0550 | No |
| 4 | ELN |  |  | 182 | 0.433 | -0.0922 | No |
| 5 | PDPN |  |  | 205 | 0.410 | -0.0936 | No |
| 6 | SLC1A3 |  |  | 237 | 0.388 | -0.1091 | No |
| 7 | HEY1 |  |  | 253 | 0.376 | -0.1033 | No |
| 8 | ZFP36L2 |  |  | 279 | 0.358 | -0.1126 | No |
| 9 | RAMP1 |  |  | 285 | 0.357 | -0.0943 | No |
| 10 | DDAH1 |  |  | 300 | 0.346 | -0.0892 | No |
| 11 | DOK5 |  |  | 302 | 0.346 | -0.0661 | No |
| 12 | BCAP29 |  |  | 308 | 0.343 | -0.0488 | No |
| 13 | ABHD3 |  |  | 310 | 0.343 | -0.0259 | No |
| 14 | PHGDH |  |  | 344 | 0.328 | -0.0484 | No |
| 15 | HES4 |  |  | 360 | 0.322 | -0.0465 | No |
| 16 | ID1 |  |  | 390 | 0.311 | -0.0646 | No |
| 17 | IFI27L1 |  |  | 400 | 0.308 | -0.0553 | No |
| 18 | FABP7 |  |  | 414 | 0.302 | -0.0519 | No |
| 19 | TTYH1 |  |  | 469 | 0.287 | -0.1063 | No |
| 20 | OLFM2 |  |  | 481 | 0.285 | -0.1014 | No |
| 21 | EFEMP2 |  |  | 507 | 0.280 | -0.1163 | No |
| 22 | IFITM3 |  |  | 512 | 0.279 | -0.1021 | No |
| 23 | CNN3 |  |  | 527 | 0.276 | -0.1020 | No |
| 24 | DKK3 |  |  | 549 | 0.273 | -0.1118 | No |
| 25 | GNG12 |  |  | 587 | 0.267 | -0.1441 | No |
| 26 | MAGT1 |  |  | 642 | 0.257 | -0.2007 | No |
| 27 | SCD |  |  | 688 | -0.263 | -0.2443 | Yes |
| 28 | MSMO1 |  |  | 691 | -0.267 | -0.2283 | Yes |
| 29 | VIM |  |  | 692 | -0.267 | -0.2094 | Yes |
| 30 | CA12 |  |  | 698 | -0.274 | -0.1970 | Yes |
| 31 | ID4 |  |  | 708 | -0.285 | -0.1893 | Yes |
| 32 | MYO10 |  |  | 710 | -0.305 | -0.1691 | Yes |
| 33 | HSPB1 |  |  | 730 | -0.378 | -0.1687 | Yes |
| 34 | IDI1 |  |  | 734 | -0.397 | -0.1448 | Yes |
| 35 | HMGCS1 |  |  | 739 | -0.418 | -0.1208 | Yes |
| 36 | DBI |  |  | 740 | -0.422 | -0.0910 | Yes |
| 37 | FABP5 |  |  | 759 | -1.641 | -0.0000 | Yes |
Table: GSEA details [plain text format]

  

Fig 2: RICHARDS\_ZHONG\_NATURE\_2018\_ASTROCYTES\_UPREG: Random ES distribution      
 Gene set null distribution of ES for **RICHARDS\_ZHONG\_NATURE\_2018\_ASTROCYTES\_UPREG**
